# Methanolic Extract from Sea Cucumber, *Holothuria scabra*, Induces Apoptosis and Suppresses Metastasis of PC3 Prostate Cancer Cells Modulated by MAPK Signaling Pathway

**DOI:** 10.4014/jmb.2103.03034

**Published:** 2021-04-30

**Authors:** Kanta Pranweerapaiboon, Kunwadee Noonong, Somjai Apisawetakan, Prasert Sobhon, Kulathida Chaithirayanon

**Affiliations:** 1 Department of Anatomy, Faculty of Science, Mahidol University, Bangkok 10400, Thailand; 2 School of Allied Health Sciences, Walailak University, Nakhonsithammarat 80161, Thailand; 3 Department of Anatomy, Faculty of Medicine, Srinakharinwirot University, Bangkok 10110, Thailand

**Keywords:** *Holothuria scabra*, apoptosis, metastasis, ROS, prostate cancer, MAPK

## Abstract

Sea cucumber, *Holothuria scabra*, is a well-known traditional Asian medicine that has been used for suppressing inflammation, promoting wound healing, and improving immunity. Moreover, previous studies demonstrated that the extract from *H. scabra* contains many bioactive compounds with potent inhibitory effect on tumor cell survival and progression. However, the effect of the methanolic extract from the body wall of *H. scabra* (BWMT) on human prostate cancer cells has not yet been investigated. In this study, we aimed to investigate the effects and underlying mechanism of BWMT on prostate cancer cell viability and metastasis. BWMT was obtained by maceration with methanol. The effect of BWMT on cell viability was assessed by MTT and colony formation assays. The intracellular ROS accumulation was evaluated using a DCFH-DA fluorescence probe. Hoechst 33342 staining and Annexin V-FITC/PI staining were used to examine the apoptotic-inducing effect of the extract. A transwell migration assay was performed to determine the anti-metastasis effect. BWMT significantly reduced cell viability and triggered cellular apoptosis by accumulating intracellular ROS resulting in the upregulation of JNK and p38 signaling pathways. In addition, BWMT also inhibited the invasion of PC3 cells by downregulating MMP-2/-9 expression via the ERK pathway. Consequently, our study provides BWMT from *H. scabra* as a putative therapeutic agent that could be applicable against prostate cancer progression.

## Introduction

Prostate cancer is the second-leading cause of cancer-related deaths in males [1]. After androgen deprivation therapy, prostate cancer inevitably develops into castration-resistant prostate cancer (CRPC) which eventually metastasizes to many vital organs, including bone, brain, liver, and lung [2, 3]. Although there are numerous chemotherapeutic agents being used against the advanced stage of prostate cancer, most patients usually end up in resistance to these drugs which results in poor prognosis and death [2]. One way to circumvent this undesirable outcome is using functional foods or nutraceuticals as anti-CRPC agents.

Cancer cells possess specific characteristics that allow them to survive. These are uncontrolled growth, abilities to avoid apoptosis, and metastasis to various other organs [4]. Reactive oxygen species (ROS) are key molecules that trigger various signaling transduction pathways [5, 6]. Excessive ROS generation causes oxidative stress and damage to a vital organelle in the cells which leads to cellular apoptosis [5]. Additionally, ROS also plays an important role in mediating tumor cell migration and invasion [7]. ROS can act in cooperation with MAPK pathway, especially JNK and p38 [8]. Therefore, natural products or nutraceutics that can result in apoptosis and inhibition of metastasis via the ROS axis may be an alternative treatment against cancer cells [9].

More than half of modern chemotherapeutic drugs used for cancer treatment are developed or derived from natural products [4]. Marine animals are currently considered as a tremendous source of bioactive compounds which have a wide range of biological properties, including anti-oxidant, anti-bacteria, anti-inflammation, and anti-tumor [10, 11]. Sea cucumber, *Holothuria scabra*, especially its body wall, is a nutritious echinoderm. Previous reports demonstrated that it contains a variety of pharmacologically active molecules, particularly triterpene glycosides [12]. This class of molecule strongly inhibits tumor progression via different underlying mechanisms including anti-proliferation, anti-angiogenesis, anti-migration, and apoptosis induction [12, 13]. Preceding reports focused on only a few types of cancers, such as breast cancer, pancreatic cancer, and leukemia [13, 14]. Although certain studies have revealed the cytotoxic effect of the sea cucumber extracts on prostate cancer cells, a possible molecular mechanism on prostate cancer cells is not fully understood yet. In this context, we explored the pharmacological effect of the methanolic extract from body wall of *H. scabra* (BWMT) on the highly aggressive human prostate cancer cell line PC3 as a future therapeutic approach in cancer progression along with a possible underlying molecular mechanism.

## Materials and Methods

### Preparation of Methanolic Extract from the Sea Cucumber, *H. scabra*

Sea cucumber, *Holothuria scabra*, was collected and identified by Prachuap Khiri Khan Coastal Fisheries Research and Development Center, Klong Wan, Prachuap Khiri Khan, Thailand. The body wall and viscera of these sea cucumbers were dissected and separated from each other. The body walls were cut into small pieces, lyophilized, and then kept at -20°C until processing. The dried specimens were extracted three times by maceration with methanol (MeOH) for 24 h each, and then concentrated under low pressure to yield the methanolic extract (BWMT). The study protocol was approved by the Faculty of Science, Mahidol University-Institutional Animal Care and Use Committee (MUSC-IACUC), No. MUSC63-002-510.

### Cell Culture

Human prostate cancer cell line (PC3) was purchased from American Type Culture Collection (ATCC, USA). The cells were cultured in RPMI-1640 medium supplemented with 10% fetal bovine serum and 1% penicillin/streptomycin (Gibco, USA). The cells were maintained at 37°C in 5% CO_2_ incubator and monitored daily. Cultured medium was changed 2-3 times per week.

### Cell Viability Assay

Cells were seeded in 96-well plates at a concentration of 1 × 10^4^ cells/well and incubated for 24 h. The cells were then treated with the sea cucumber extracts in various concentrations ranging from 1 to 100 μg/ml. After 24 and 48 h treatments, MTT (3-(4,5-dimethylthiazol-2-yl)-2,5-diphenyltetrazolium bromide) (Sigma-Aldrich, USA) assay was used to analyze the effect of BWMT on cell viability. Ten microliters of MTT (5 mg/ml) was added and incubated at 37°C in 5% CO_2_ for 3 h. At the indicated times, supernatant was discarded. Then, 100 μl of 0.1 M hydrochloric acid (HCl) in isopropanol was added to dissolve the formazan crystal. The absorbance of each well was read on a microplate reader (ELx808, Biotek Instruments Inc.,USA) at 562 nm. The percentage of cell viability was calculated and compared with the untreated group.

### Colony Formation Assay

Colony formation assay was performed to assess the long-term cytotoxic effect of the extracts on PC3. Cells were treated with the extracts at indicated concentrations for 6 h. Subsequently, the treated cells were detached by trypsin. The treated prostate cancer cells were counted at a concentration of 1 × 10^3^ cells/well and then seeded in 24-well plates to observe the novel colony formation for 14 days. The culture medium was replaced every 3 days. The colonies were fixed in methanol for 10 min, stained with crystal violet, and then observed under a light microscope.

### Measurement of Intracellular Reactive Oxygen Species (ROS)

Dichloro-dihydro-fluorescein diacetate (DCFH-DA) staining, which is a colorimetric and fluorometric probe, was used to detect the level of ROS. Prostate cancer cells were plated in a black, flat-bottomed 96-well microplate at a concentration of 1×10^4^ cells/well and incubated overnight in a CO_2_ incubator. Culture medium in the plate was discarded. Cells were washed with phosphate-buffered saline (PBS) (Gibco) to eliminate residue from the culture medium. Cells were then incubated with 5 mM DCFH-DA (Thermo Fisher Scientific, USA) in phenol red-free culture medium for 45 min. The extract was diluted in phenol red-free culture medium at indicated concentrations prior to treating the cells. The fluorescence intensity was read at an emission wavelength of 528 nm and an excitation wavelength of 485 nm (Tecan Spark 10M, Switzerland). All values were calculated as percentage fluorescent intensity relative to the control.

### Cell Cycle Analysis

To analyze the effect of BWMT on cell cycle phase distribution, PC3 cells were plated in 6-well plates at a concentration of 3×10^5^ cells/well. Cells were treated with various concentrations of the extracts for 24 h. Subsequently, cells were harvested and tested with the BD Cycletest Plus DNA Reagent Kit (BD Biosciences, USA). In brief, trypsin buffer was added and incubated for 10 min at room temperature followed by the addition of trypsin inhibitor and RNase buffer. Cold PI solution was added and incubated on ice for 10 min. The stained cells were analyzed by BD FACSCalibur flow cytometer (BD Biosciences, Germany). The percentage of cell cycle distribution was calculated and compared with untreated cells.

### Hoechst Staining

Hoechst staining was performed to investigate the effect of BWMT on apoptosis induction. PC3 cells were seeded in 6-well plates at a concentration of 1 × 10^5^ cells/well and incubated with the extracts at indicated concentrations for 24 h. After treatment, the supernatant was removed and washed twice with PBS. Cells were fixed with 4% paraformaldehyde and then incubated with Hoechst 33342 (Abcam, UK). The cells were observed and images were recorded under a fluorescence microscope (BX53, Nikon, Japan).

### Annexin V/Propidium Iodide Assay

The effect of the extracts on prostate cancer cell apoptosis was determined by FITC Annexin V Apoptosis Detection Kit I (BD Pharmingen Inc., USA). PC3 cells were plated in 6-well plates at a seeding density of 1 × 10^5^ cells/well and incubated for 24 h. Subsequently, cells were treated with BWMT at indicated concentrations for 24 h. After incubation, each cell solution was transferred to a fresh flow cytometry tube. Five microliters of Annexin V-FITC and PI were added. Next, cells were incubated at room temperature in dark condition for 15 min. The stained cells were analyzed by BD FACSCalibur flow cytometer (BD Biosciences). The percentage of apoptotic cells was calculated and compared with untreated cells.

### Transwell Assay

The Corning HTS Transwell-24 well (Corning, USA) was used to examine the effect of BWMT on prostate cancer cell migration and metastasis. The lower chambers were filled up with 10% FBS in culture medium. The upper chambers were coated with 20 μl Matrigel (Corning). Cells were treated with the sea cucumber extract which was diluted in serum-free medium and placed in the upper chambers. The system was incubated in the CO_2_ incubator for 24 h. The lower side of the membranes containing migrating and metastasis cells was fixed with methanol for 10 min. Afterward, the membranes were stained with crystal violet. The migrating/metastatic cells were assessed under a microscope. The percentage of migrating/metastatic cells was calculated and compared with untreated cells.

### Western Blotting Analysis

After 24 h of treatment, whole protein of each treated group was harvested with 1 mM phenylmethylsulfonyl fluoride (PMSF) in RIPA buffer (Cell Signaling Technology, USA). The extracted proteins were centrifuged at 14,000 ×*g* in a cold microfuge for 10 min. The supernatants were collected for further experiments. The total protein concentration was determined by using a BCA Protein Assay Kit (Thermo Fisher Scientific). For each treatment, 30 μg of crude protein was separated by 12% sodium dodecyl sulfate-polyacrylamide gel electrophoresis (SDS-PAGE) and transferred onto nitrocellulose membranes. Shortly afterward, the membranes were blocked with 5% skim milk in Tris-buffered saline with 0.1% Tween 20 detergent (TBS-T) (Gibco) for 3 h. The blots were incubated overnight at 4°C with primary antibodies against MMP-2, MMP-9, Bcl-2, BAX, Caspase3, JNK, p-JNK, p38, and phospho-p38 (Cell Signaling Technology). After washing with TBS-T, the blot membranes were then incubated with mouse anti-rabbit HRP antibody for 2 h. Chemiluminescent signal was visualized by using an ECL Chemiluminescent Substrate Reagent Kit (Thermo Fisher Scientific). Band intensity was determined by ImageJ software. β-Actin was used as an internal control.

### Statistical Analysis

All data were expressed in mean ± SD from three independent experiments and then analyzed using Prism6 (GraphPad Software, USA). Statistical comparisons were performed using one- or two-way analysis of variance (ANOVA) with the Bonferroni multiple comparison tests.

## Results

### BWMT Inhibited Viability of Highly Metastatic Prostate Cancer Cells

**Fig. 1:**
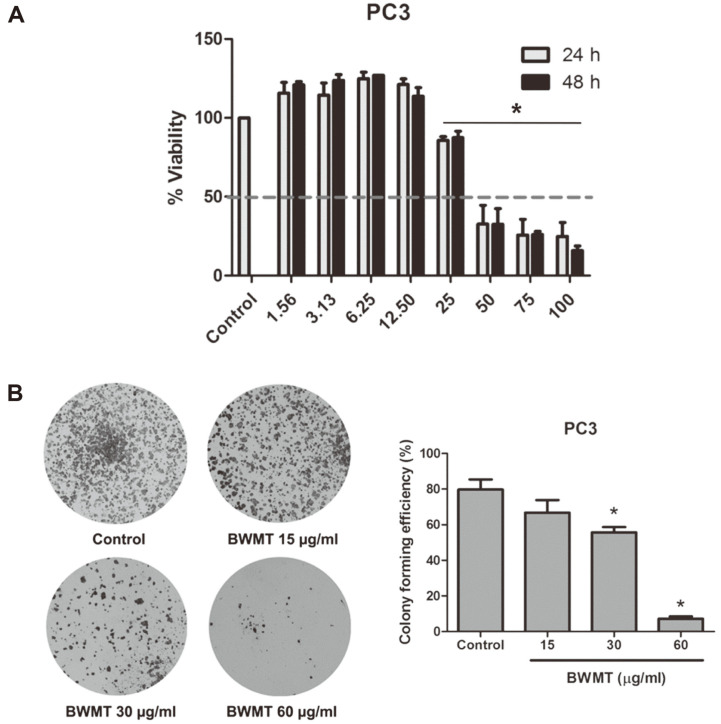
BWMT inhibits cell viability of advanced stage of prostate cancer cell line. (**A**) Cells viability was examined by MTT assay after treatment with 0-100 μg/ml BWMT for 24 and 48 h. (**B**) Cells were treated with 15, 30, and 60 μg/ml BWMT to observe the long-term cytotoxic effect (14 days) by colony formation assay. Colonies were stained with crystal violet and observed under a microscope. The bar graph is presented as mean ± SD. *p < 0.05, significant difference compared with control group. The experiments were performed independently for 3 times.

To examine the possible cytotoxic potency of BWMT on prostate cancer cells, PC3 cells were exposed to 0-100 μg/ml BWMT for 24 and 48 h. MTT assay was performed to determine cell growth inhibition. As shown in Fig. 1A, BWMT exhibited cytotoxic effect against PC3 cells in a concentration-dependent manner. No significant changes were observed between 24 and 48 h treatments. BWMT at concentrations 50-100 μg/ml could inhibit the viability of PC3 cells when compared with the non-treated group. In contrast to the high concentrations, BWMT at 1.56-12.5 μg/ml BWMT mildly promoted the proliferation of PC3 cells. The IC_50_ of BWMT against PC3 was 28.47 ± 0.04 and 30.73 ± 0.03 μg/ml at 24 and 48 h of treatments, respectively.

Additionally, the long-term cytotoxic effect of BWMT on prostate cancer cells was also determined by the colony formation assay over a period of 14 days. The concentrations of BWMT used in this and further experiment were designated based on the IC_50_ values of each group of cells. Therefore, PC3 cells were incubated with 15, 30, and 60 μg/ml BWMT. The colony-forming efficiency graphs revealed a significant decrease in numbers of colony formation by PC3 cells after BWMT treatments in a concentration-dependent manner in comparison to the control group (Fig. 1B). Taken together, these findings indicated that BWMT has potential in inhibiting the growth of these highly metastatic prostate cancer cells.

### BWMT Promoted Intracellular ROS Accumulation in Prostate Cancer Cells

**Fig. 2:**
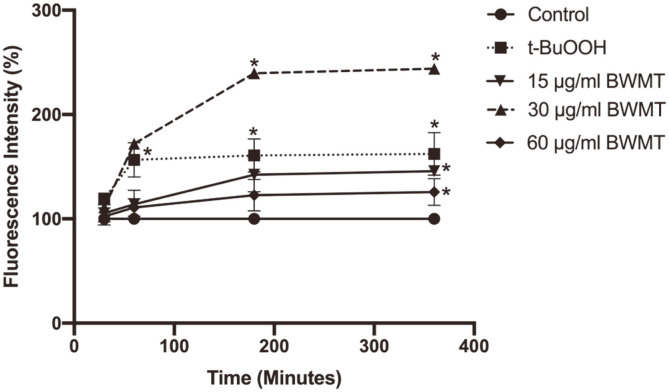
BWMT promoted intracellular ROS accumulation in PC3 cells. The amount of ROS generation in each group was calculated as percentage of fluorescence intensity relative to the control. Data are expressed as mean ± SD from three independent experiments (*p < 0.05).

We further investigated the mechanism underlying cytotoxicity of the BWMT on prostate cancer cells. Previous studies demonstrated that the compounds extracted from *Stichopus japonicus* and *Holothuria parva* stimulated colorectal cancer and melanoma to generate an excessive amount of intracellular ROS [15, 16]. Here, we also found increased intracellular ROS in PC3 cells treated with BWMT compared with control cells and those treated with 250 μM tert-Butyl hydroperoxide (t-BuOOH) (Fig. 2). At 60 min, t-BuOOH significantly induced intracellular ROS production at 156.5 ± 6.42% (*p* < 0.05) and this level was maintained until the end of the experiment. Likewise, the ROS in PC3 cells treated with 30 μg/ml BWMT was sharply increased up to 239.62 ± 1.92% at 180 min (*p* < 0.05). This suggested that the cytotoxic effect of BWMT on PC3 cells might be mediated by ROS-induced cell death.

### BWMT Triggered Cell Cycle Arrest and Cellular Apoptosis in Prostate Cancer Cells

**Fig. 3:**
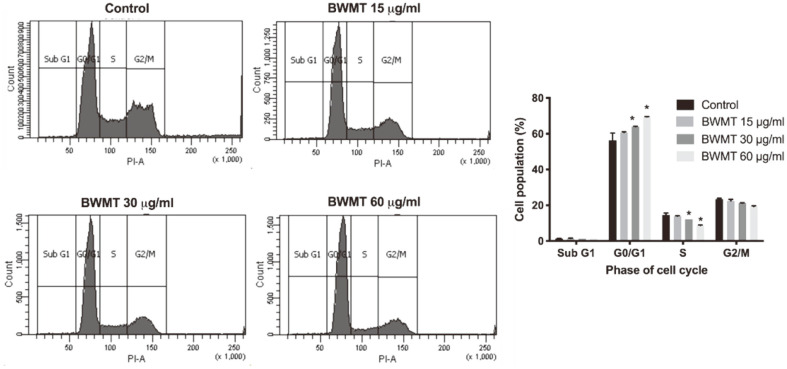
The cell cycle distributions of PC3 after treatment with the designated concentrations of BWMT. Data were expressed as mean ± SD from three experiments (*p < 0.05).

Apart from cytotoxic effect, a high level of ROS initiates cell cycle arrest and apoptosis [17]. To investigate whether BWMT altered the cell cycle of prostate cancer, the treated cells were stained with PI/RNase and the cell cycle phases were detected by flow cytometer. As shown in Fig. 3, after treatment with 30 and 60 μg/ml BWMT, the proportion of cells in G_0_/G_1_ phase significantly increased (*p* < 0.05), while the proportion of cells in the S phase was reduced compared to the control group. Therefore, BWMT interfered with the proliferative cycle of prostate cancer cells.

**Fig. 4:**
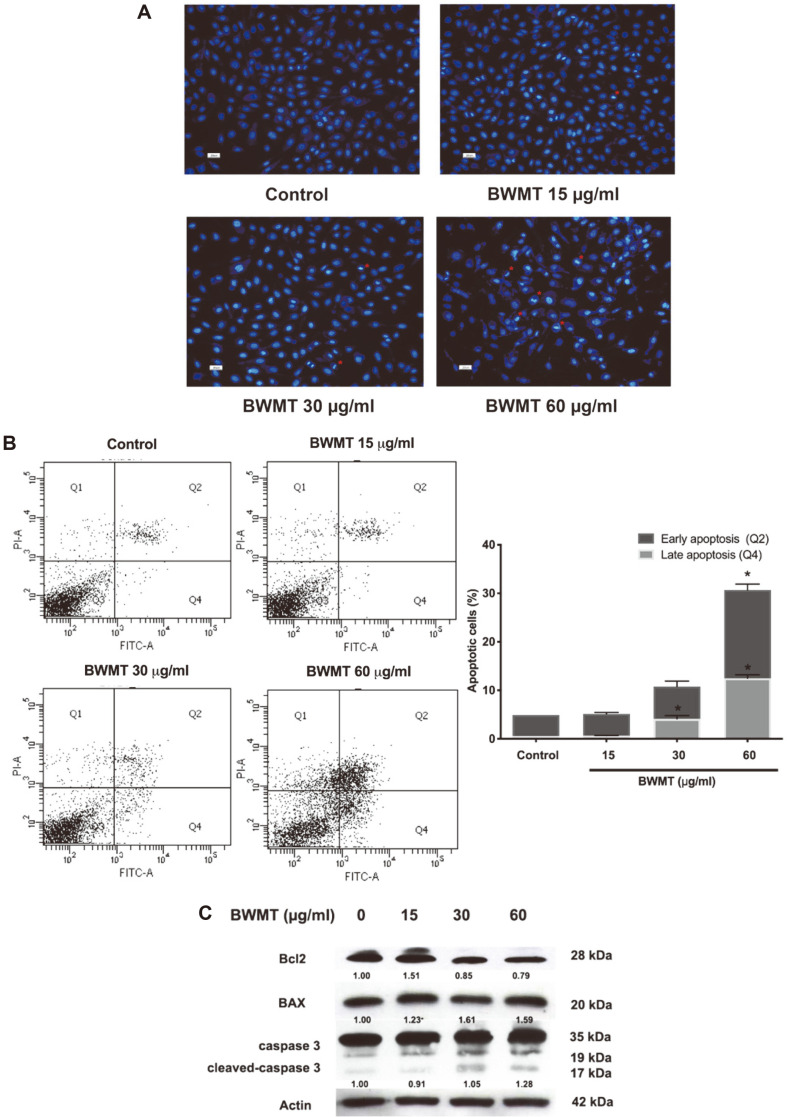
The effects of BWMT on apoptosis induction in prostate cancer cells. (**A**) Nuclear morphology of Hoechst 33342 staining nuclei of BWMT-treated prostate cancer cells. Asterisks indicate nuclei condensation. (**B**) Rate of PC3 apoptosis analyzed by Annexin V-FITC/PI dual-labeling and flow cytometry. (**C**) Western blotting evaluated the effect of BWMT on apoptotic markers expressions. Data were expressed as mean ± SD from three experiments (*p < 0.05).

To further explore the mechanism of BWMT on apoptotic induction, the treated prostate cancer cells were stained with Hoechst 33342 and visualized under a fluorescence microscope at 20X magnification. The Hoechst staining revealed the increased number of condensed nuclei in the prostate cancer cells after treatment with BWMT (Fig. 4A). This was also consistent with the data analyzed by Annexin V-FITC/PI dual-labeling (Fig. 4B). Apoptotic cells significantly increased after exposure to 30 and 60 μg/ml BWMT from 4.9 ± 1.25% in the control group to 10.8 ± 1.8%, 32.2 ± 4.2%, respectively. Western blotting analysis of the expressions of apoptotic markers also demonstrated that BWMT reduced the expression of anti-apoptotic Bcl2 and enhanced the expression of pro-apoptotic protein BAX and cleaved caspase-3 (Fig. 4C). These data indicated that BWMT promotes cell cycle arrest and induces apoptosis pathway.

### BWMT Inhibited Prostate Cancer Cell Migration and Invasion

**Fig. 5:**
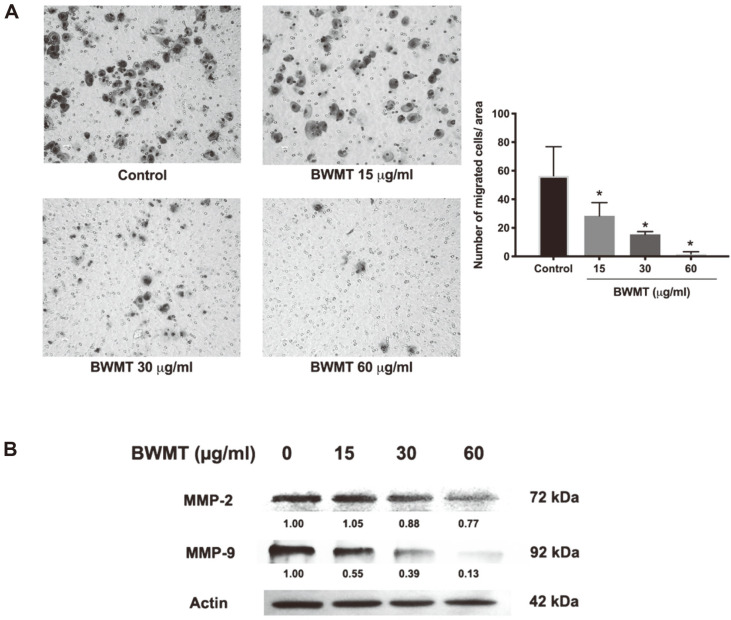
Inhibition of migration and invasion of prostate cancer cells by BWMT. (**A**) PC3 cells were treated with the designated concentrations of BWMT and placed on an upper chamber coated with Matrigel. Cells that migrated and invaded to the lower chamber were stained with crystal violet and observed under a microscope. (**B**) The western blot revealed the effects of BWMT on the expressions of invasion markers MMP-2 and MMP-9. Data were expressed as mean ± SD from three experiments (*p < 0.05).

Migration and invasion are critical steps in the spread of cancer cells, which is the major cause of prostate cancer-related death [3]. To determine the effect of BWMT on prostate cancer migration, PC3 cells were treated with the designated concentration and evaluated with the transwell migration assay. BWMT significantly inhibited the migration and invasion of PC3 (Fig. 5A). There was 56.50 ±10.14% of migrated cells from the untreated cells in the lower chamber, while 15, 30, and 60 μg/ml BWMT-treated groups were 28.50 ± 13.89%, 15.50 ± 0.96%, and 1.75 ± 0.75%, respectively. In addition, BWMT decreased metalloproteinase-2 and -9 (MMP-2 and MMP-9) expressions in a concentration-dependent manner (Fig. 5B). Altogether, the results implied that BWMT possessed a strong inhibition on prostate cancer cell migration and invasion.

### BWMT Mediated MAPK Signaling Pathway in Prostate Cancer Cells

**Fig. 6:**
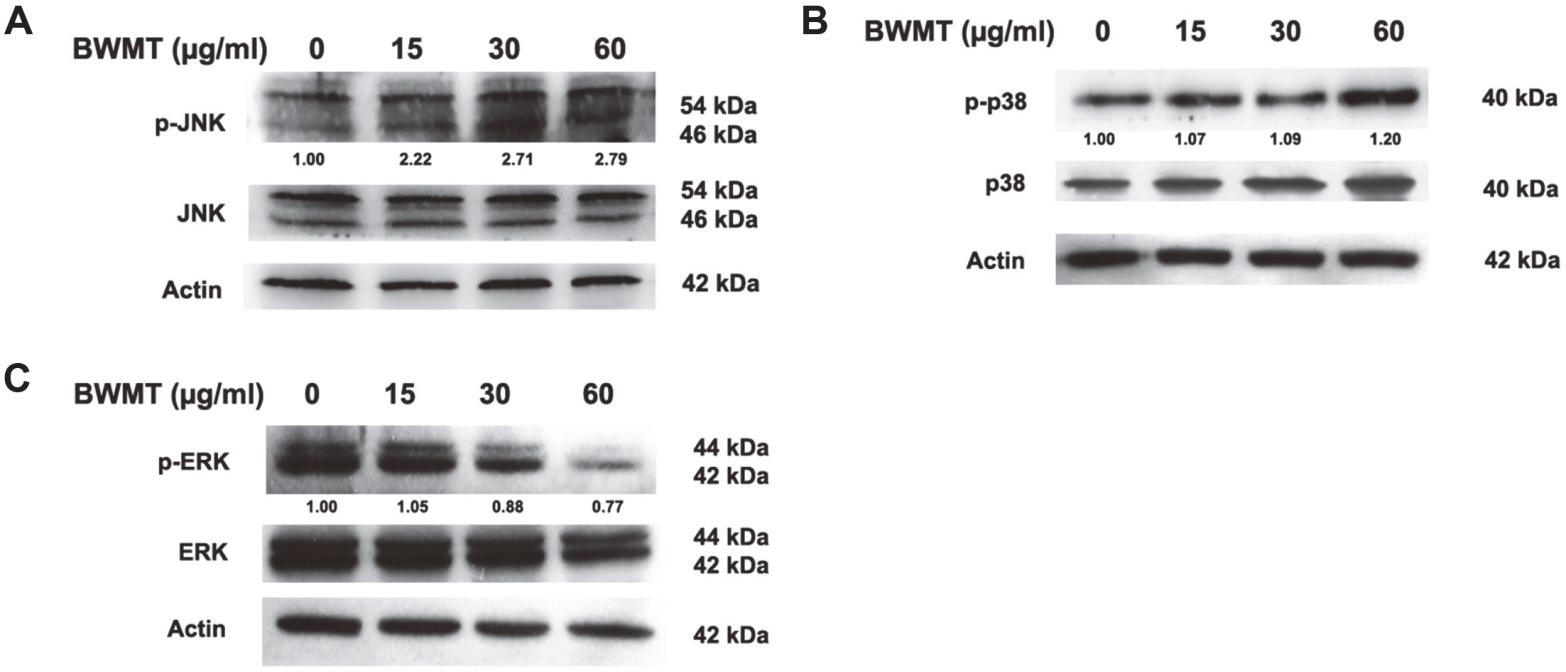
The effects of BWMT on MAPK pathways (A) JNK, (B) p38, and (C) ERK. The band intensities of p-JNK, pp38, and p-ERK were normalizing with the JNK, p38, and ERK from three experiments.

Since BWMT exhibited its effects on prostate cancer cells by enhancing the level of ROS, resulting in inducing cell cycle arrest and apoptosis, as well as suppressing cell migration and invasion, the underlying mechanism was further investigated. Several studies demonstrated that the MAPK pathways, especially JNK and p38, are responsible for ROS-related cellular senescence [8, 18]. As shown in Fig. 6, BWMT could not alter the level of JNK and p38 expression, but their phosphorylation forms were significantly increased (*p* < 0.05). By contrast, the level of p-ERK was abolished in BWMT-treated groups. Therefore, these data demonstrated that JNK and p38 contributed to the cytotoxic effects of BWMT on prostate cancer cells.

## Discussion

Prostate cancer is the second-leading cause of cancer-related death in males [9]. Therapeutic resistance and rapid metastasis are the main causes of the high mortality rate of prostate cancer [1]. Thus, recent research has focused on discovering novel molecules from natural products that overcome these intransigent behaviors of prostate cancers [17]. So far, sea cucumber-derived natural products have attracted much attention due to their wide ranges of pharmacological activities, including the inhibitory effects on cancer cell proliferation, migration, invasion, and angiogenesis [11, 14, 19]. Triterpene glycosides are the signature secondary metabolites isolated from holothurians [20] that play essential roles in mediating cytotoxicity in various cancers [21]. Our previous report revealed that a high yield of triterpene glycosides could be observed in the alcoholic extract from *H. scabra* and have a high potential to inhibit tumor growth via inducing apoptosis in glioblastoma and breast cancer cells [13, 22], as well as disrupting the aerobic glycolysis in breast cancer cells [13]. In this study, we have further clarified a similar inhibitory effect of BWMT on the induction of apoptosis and inhibition of metastasis in a aggressive and metastatic phenotype of human prostate cancer cell line (PC3) through MAPK signaling pathway.

To better understand how BWMT suppresses prostate cancer cell survival, our experiments were designed to explain the effect of BWMT on ROS production and activities. Reactive oxygen species serve as a secondary messenger that regulate several physiological processes. Generally, the mitochondria are the primary source of ROS generation and are prone to become the main target of oxidative damage. Our data demonstrated that BWMT could stimulate ROS production in PC3 cells. This evidence is highly consistent with the effects of methanolic extracts of the sea cucumber *H. oculata* and *S. japonicus* that markedly induced ROS generation in hepatocellular carcinoma and colorectal cancer cells [15, 23]. It has been suggested that prolonged exposure of cells to ROS enhanced cell cycle arrest at G1 phase due to the defensive mechanism of cells against oxidative damage [24]. Thus, the cell cycle of PC3 cells was arrested at G_0_/G_1_ phase after exposure to BWMT. These data were similar to the effect of the extracts from *H. atra* that activated cell cycle arrest at G_0_/G_1_ phase in hepatocellular carcinoma [25]. The induction of cell cycle arrest at the early stages like the G_0_/G_1_ phase is accountable for the high anti-proliferative potential of compounds and related caspase-dependent cell death [26, 27].

Oxidative stress can trigger mitochondrial dysfunction leading to conformational changes of Bcl-2-associated X and apoptotic regulator BAX proteins. Then, cytochrome C is released into the cytoplasm which activates the downstream caspase cascades, and ultimately facilitating cell apoptosis [15, 23]. After treatment with BWMT, the flow cytometry detected Annexin V-FITC/PI-labeled cells, confirming that PC3 cells shifted their fate to apoptosis with nuclear condensation and fragmentation. BWMT also affected apoptotic markers by abolishing the level of Bcl2 and upregulating BAX and caspase-3 expressions. This implied that BWMT is able to induce prostate cancer cell apoptosis.

Finding compounds that can induce cancer cell apoptosis and metastasis is one of the promising strategies to treat drug-resistant cancers [9]. MMP-2 and MMP-9 are important enzymes responsible for degradation of the extracellular matrix, resulting in cancer cell migration and invasion [28]. In this experiment, we proved that BWMT inhibited prostate cancer cell migration and invasion by reducing MMP-2/-9 protein expressions. Consistent with the report of TBL-12, the commercial sea cucumber extracts could prevent prostate cancer metastasis [12]. Literally, the mitogen-activated protein kinases (MAPKs) are serine-threonine protein kinases consisting of growth factor-regulated extracellular signal-related kinases (ERKs), and the stress-activated MAPKs, c-Jun NH_2_-terminal kinases (JNKs) and p38 MAPKs. All have been deciphered as having specific roles in cancer initiation, growth, and metastasis [29]. The upregulation of ERK is often mediated by the growth factor stimulation, resulting in tumor invasion and metastasis [30]. Disruption of ERK expression inhibits the invasive cancer cells [31, 32], whereas highly activation of JNK/p38 correlated with the oxidative stress environment is critical for apoptosis induction which can be observed in various cancers, including glioblastoma and gastric cancer [18, 33-35]. On similar lines, this idea is reinforced by the manner in which BWMT treatment effectively induces apoptosis in PC3 cells via ROS-mediated p38/JNK-MAPK signaling pathway. Moreover, the suppression of ERK pathway by BWMT downregulates the MMP-2/-9 protein expressions, thereby inhibiting the invasion of metastatic PC3 cells.

## Conclusion

Our findings showed, for the first time, the strong potential of methanolic extract from the body wall sea cucumber extract (BWMT) in inhibiting the progression of the advanced-stage prostate cancer cell line PC3 by inducing apoptosis and inhibiting metastasis via the enhancement of intracellular ROS level through activation of JNK/p38 and downregulation of ERK pathways, respectively. Therefore, BWMT may be a valuable adjuvant therapy for advanced-stage prostate cancer treatment.
